# Canadian Men’s perspectives about active surveillance in prostate cancer: need for guidance and resources

**DOI:** 10.1186/s12894-017-0290-7

**Published:** 2017-10-27

**Authors:** Margaret Fitch, Kittie Pang, Veronique Ouellet, Carmen Loiselle, Shabbir Alibhai, Simone Chevalier, Darrel E. Drachenberg, Antonio Finelli, Jean-Baptiste Lattouf, Simon Sutcliffe, Alan So, Simon Tanguay, Fred Saad, Anne-Marie Mes-Masson

**Affiliations:** 1 Sunnybrook Health Sciences Centre, 2075 Bayview Ave, Toronto, ON Canada; 2 Institut du cancer de Montréal and Centre de recherche du Centre hospitalier de l’Université de Montréal, 900 St Denis St, Montreal, QC Canada; 3 McGill University and McGill University Health Centre, 1001 Decarie Blvd, Montreal, QC Canada; 4 University Health Network, 610 University Ave, Toronto, ON Canada; 5 Manitoba Prostate Centre, 675 McDermot Ave, Winnipeg, MB Canada; 6 Department of Surgery Université de Montréal, 2900 Edouard Montpetit Blvd, Montreal, QC Canada; 7 Terrry Fox Research Institute, 675 West 10th Avenue, Vancouver, BC Canada; 8 Vancouver Prostate Centre, 2660 Oak St, Vancouver, BC Canada; 9 Department of Medicine, Université de Montréal, 2900 Edouard Montpetit Blvd, Montreal, QC Canada

**Keywords:** Active surveillance, Prostate cancer, Decisions making, Focus group, Low risk disease

## Abstract

**Background:**

In prostate cancer, men diagnosed with low risk disease may be monitored through an active surveillance. This research explored the perspectives of men with prostate cancer regarding their decision-making process for active surveillance to identify factors that influence their decision and assist health professionals in having conversations about this option.

**Methods:**

Focus group interviews (*n* = 7) were held in several Canadian cities with men (*N* = 52) diagnosed with prostate cancer and eligible for active surveillance. The men’s viewpoints were captured regarding their understanding of active surveillance, the factors that influenced their decision, and their experience with the approach. A content and theme analysis was performed on the verbatim transcripts from the sessions.

**Results:**

Patients described their concerns of living with their disease without intervention, but were reassured by the close monitoring under AS while avoiding harmful side effects associated with treatments. Conversations with their doctor and how AS was described were cited as key influences in their decision, in addition to availability of information on treatment options, distrust in the health system, personality, experiences and opinions of others, and personal perspectives on quality of life.

**Conclusions:**

Men require a thorough explanation on AS as a safe and valid option, as well as guidance towards supportive resources in their decision-making.

## Background

Prostate cancer, the most commonly diagnosed cancer in Canadian men, accounted for 21,600 new cancer diagnoses in 2016 (Canadian Cancer Statistics, 2016). PCa consumes a significant amount of treatment resources [1], initiating efforts to distinguish men with high risk disease who require therapeutic intervention to avert premature death and disability. For men with low risk disease, interventional therapy is neither required nor appropriate to ensure a lifespan uncompromised by cancer or by the therapeutic consequences (reviewed in [2]).

Presently, men with low risk PCa are given the option of ‘active surveillance’ (AS). With AS, practitioners delay curative treatment until there are indications that the disease is progressing. Very low- or low-risk localized PCa is defined to include tumor stage (T1c, prostate-specific antigen [PSA] detected or T2a, small palpable nodule); PSA value (less than 10 ng/mL); Gleason score (≤ 6); and extent of disease in biopsy (<3 biopsy cores positive and ≤50% cancer in any cores) [3] (reviewed in [4, 5]). With the advent of PSA screening, more low risk cancers have been detected along with recommendations of AS.

Patients and practitioners have expressed discomfort with the option of AS as a significant proportion of patients may be understaged given the intrinsic sampling error of prostate biopsies [6]. Investigation is underway to identify biomarkers that accurately detect truly low risk disease for patients with a grade 6 cancer or even those with Gleason score 7 [1, 7]. This would enlarge the pool of individuals for whom AS would be considered an appropriate approach.

The Canadian PCa Biomarker Network team investigated the uptake of AS in Canada in 2010 [8]. AS is widely practiced across Canada, but important regional differences exist in its use for reasons that remain unknown. Additionally, there is little systematic understanding about the factors that influence the acceptance of and adherence to AS in PCa in Canada.

The uptake of AS is dependent upon patient, clinical, and societal factors that influence the individual’s decision about a regimen of AS. While prevailing public messages about cancer advocate immediate treatment, AS infers that curative treatment is applied only when the disease progresses to a clinically significant stage. Thus, men who consider AS must decide if they can live with their disease without receiving any treatment or intervention at all, or for some time.

## Methods

This research explored the perspectives of men with PCa regarding their decision-making process for AS. Understanding men’s perspectives on following a regimen of AS would help clinicians in providing clear information and meaningful discussions on treatment options and supportive care.

The study utilized a qualitative descriptive design [9] and recruited men from PCa programs in Montreal, Toronto, Winnipeg, Vancouver, and Thunder Bay. These programs provided access to urologists, medical oncologists and radiation oncologists. Candidates for AS had low grade, localized PCa and were informed about the study by a clinician providing their care. A local research coordinator contacted and informed interested individuals on study details and the arrangements for participation upon consent. Research ethics approval was obtained from each of the participating sites including the Centre de recherche du centre hospitalier de l’Université de Montréal, the McGill University Health Centre, Cancer Care Manitoba, the University Health Network and the University of British Columbia.

Participants either attended one focus group or engaged in a single interview if they were unable to attend the group session. The focus groups and interviews were facilitated by qualified personnel and were conducted in the language preferred by the participants (MF all English, CL all French). All sessions were attended by three other team members who assisted with the logistics, note-taking, and clarification of questions from the participants. Sessions and interviews were audio-recorded and transcribed verbatim.

Focus groups and interview guides were developed for the study and covered the same topic. Participants were asked to describe their experiences in being diagnosed, hearing about AS, deciding their course of action, communicating their decision with others, seeking information, being on AS, and deciding whether to stay on the regime or not. Questions were open-ended and probes were inserted only for clarification purposes.

### Analysis

The transcripts were subjected to a qualitative description analysis [9]. Four team members (MF, KP, AMM, VO) read transcripts independently, taking marginal notes about the content. Team members shared their perspectives on all content identified and designed a content-coding framework based on shared perspectives. The content-coding framework contained a list of topics and definitions regarding what type of data belonged in each of those topic categories. Two members (MF, KP) used this framework to code all transcripts from the focus groups and interviews, and then reviewed the coded categories in-depth before summarizing the content for each category with an identified key theme (i.e., commonly held perspectives by the participants). The analysis was presented to three other team members who reviewed the clarity and relevance of the findings (two team members had attended the group sessions while the other was a clinician who interacted with the men considering AS). The resulting consensus about the findings provided the basis for this manuscript which focuses on decision-making about AS.

## Results

### Patient characteristics

Seven focus groups were held with locations in Montreal (one English and one French), Toronto (two groups), Winnipeg, Vancouver, and Thunder Bay. Nine men engaged in one-on-one interviews. A total of 52 men participated, ranging in age from 53 to 81 years (mean = 67.8; median = 68). Among them, 70.8% had completed post-secondary education or above. Participants with PCa had been living with their disease since diagnosis for 1 to 16 years (median = 3). Table 1 presents the number of men who were on, or had been on an AS regime. Overall, the participants who had been on AS reflected an average of 3.4 years of experience with the approach (range of 1 to 15 years). These data were self-reported by men on the demographic questionnaire.

**Table 1:** Participant Characteristics

Treatment decision-making stage	Number of participants (out of 52)
Diagnosed with prostate cancer and presently under active surveillance	38
Diagnosed with prostate cancer and withdrew from active surveillance	
-for reasons of disease progression -for reasons other than disease progression	0 1
Diagnosed with prostate cancer and decided to undergo surgery or radiation at the beginning	9
Diagnosed with prostate cancer and currently deciding about treatment	2
Declined to provide data	2

### Perspectives shared by participants

Four main themes emerged from the analysis of data. Each are described below, drawing from relevant content categories and illustrated by patient quotes in Table 2. The findings captured a range of perspectives from men on being diagnosed with PCa, seeking relevant information and communication, and making decisions on their course of action. Participants varied in their viewpoints and understandings about AS. Factors that influenced their decision-making on AS have implications for clinical practice.

**Table 2:** Representatives quotes from main theme content categories

Main Theme	Contributing content categories	Illustrative quote examples
An important decision is needed at a time of emotional upset and uncertainty	Obtaining a definite diagnosis can take time	*I had a physical with my family physician and he ordered a PSA test. The test revealed a slightly higher level…I was referred. I think I went through a number of PSA tests and they sort of bounced around, up and down, and eventually reached the stage where Dr. X concluded a biopsy was in order. The first biopsy revealed, out of ten samples, that eight were OK and two were inconclusive. I think we did another one or two PSA tests and it kept rising, which resulted in another biopsy. And this time one of the samples revealed cancer.*
	Hearing the definite diagnosis is a shock	*I felt I’d been hit by a truck.* *Why me? Why did this happen to me? And now? What do I do?*
	Having little information about prostate cancer causes uncertainty	*It’s amazing that you don’t really know about prostate cancer until you are diagnosed; like, why would I want to know about it?* *I really didn’t know anything about prostate cancer or even what the prostate was…I wasn’t sure what was going on.*
	Being told it is your decision to make was surprising	*He said, ‘You have to decide what you want to do.’ I was, like sort of, ‘You tell me what to do.’ You know? That’s what I found difficult…he didn’t say, ‘You definitely need this or that.’ He said, ‘Well, what did you decide?’ Me, I said, ‘Well what are the choices? What would you recommend?...What would you tell your brother?’*
Information is necessary on a number of topics before a decision can be made about active surveillance	Information is needed on prostate cancer, treatment options, expected impacts/benefits, side effects, and active surveillance	*For example, I went into the biopsy without any idea what I was going into…you should prepare people better for what’s ahead.* *I had not heard about active surveillance before at all…when I first got cancer, and before I read up on active surveillance, I must admit, well, I thought I got cancer, get it out right away. What the hell am I doing walking around with it? It never crossed my mind, I guess I never thought of something growing so slowly that it’s not going to, it will never effect you…but that’s what’s going through my mind a lot now.*
	There is a need to actively seek information	*The first thing I did when I got home was go on the Internet.* *You need to explore all the options so when push comes to shove and you need to cross that bridge, at least you are better armed to make an informed decision.* *I had really wished there had been a group like this that I could sit down and talk about making decisions. I felt quite isolated, that I had to go out and search for people.*
	There is a need to check with different sources of information	*I talked with everyone I could. It felt like I had all the time I needed to make a decision, and that I could make the best one for me. I just needed to know the options for me and the possible impacts in my situation before I made it.* *I did a ton of reading and I spoke to a ton of people who had had it. It didn’t confuse me but it didn’t necessarily help me sort if all out.*
	It is important to sort out what information is relevant to the individual’s own situation	*There is a lot of information out there, but I do not know how accurate it is.* *I had to sort out what applied to me, to my disease and situation. It was hard to know just from everything I read and had heard.*
Disease status and quality of life are important factors for men in deciding about active surveillance	Various factors are taken into consideration in making a final decision about active surveillance	*I took it all into account [the doctor, the reading, talking to my wife]…weighed all the facts, gathered my evidence, all the information, and then made an informed decision.* *For me, it was a combination of factors…weighing them out and coming to a logical conclusion.*
		Understanding of the disease and potential for harm: • *I was told it was small and low grade…a little level of cancer.* • *They told me it is mild…and that the tumor cells are slow growing…it won’t likely get me before something else.* Understanding of active surveillance and potential for benefit: • *My first reaction to the concept [active surveillance], which came right at the beginning from the urologist, that’s really doing nothing. So I am not sure that’s the right thing to do…but he laid it all out…and as I researched it, and got into it, it is doing something. It’s actually monitoring in a quite regular basis. I got comfortable with that.* • *It’s not about doing nothing, you are doing something. You are monitoring and going to catch it in time if it gets bigger. I will have time to act*. Side effects of treatment • *If I could go another 10 years without an operation, I’d feel good about that, about not having one. I’m 67 now.* • *I did a lot of reading and found a lot of negative things about the surgery.* Wanting to be rid of cancer • *As soon as I found out that I got it, I thought, ‘What the hell, I gotta get rid of this’ cause, you know, in everybody’s mind cancer isn’t good for you.* • *I said, ‘If I got it, might as well get rid of it…get it over with.’* Past experiences with family/friends • *I have a brother who is 15 years younger than me and he had his prostate removed. His cancer was small too. But he couldn’t live with it…but I can. I’m 64. Maybe if I was younger I’d have had it out too* • *My father had treatment for prostate cancer and his life was never the same again. I didn’t want that for me. I am too young.* Medical opinion • *I had four doctors that have more or less indicated to me that it is not a major thing [to wait].* • *It was my doctor’s decision. But I was very relieved that I did not have to go under the knife, you know.* • *I am beginning to fear surgery but, definitely, if the doctors all said, ‘Listen, you’ve got to get it out’, I will definitely go for surgery when the time comes, if it happens to come, I will do it.* Age and health status • *I have sort of decided that I am not going to do anything rash. I just turned 80 so it’s not as if I am in my 50’s. So I decided the best thing is just to go through with it, to do the surveillance.* • *I was more or less on [the idea that] the less aggressive form of action would be better for me right now…postpone as long as we can. Fifty-seven is too young to go under the knife.* • *I am young, I can deal with it [surgery] now and recovery will be quicker than if I wait and do something when I was in later years.* Emotional toll over time • *Any time you have a bit of an issue you would think, ah, maybe it’s because I have prostate cancer, so I decided to get it out, taken care of at that time.* • *You have to learn to live with the fact you have cancer, at least a little bit of it, and that it may never get you.* • *I decided I don’t want to live with this [prostate cancer] anymore…I am tired. So, let’s do it [treatment].* Family viewpoints • *My wife said, ‘You need to be treated. I want you to be here.’ But for me, active surveillance sounded fine.* • *But my two daughters said, ‘Dad, don’t fool around with it. Deal with it.’ There was family pressure. So I made a decision to go with radiation, stop what was there.* • *My wife was involved too…it wasn’t just my decision. We are together on that…but she wanted me to be treated!* Not enough information to decide • *I have read so much and talked to a lot of people, and the information is really not clear. That’s why I am on active surveillance. I feel I don’t have all the information [to decide about treatment].*
	Disease status is an important consideration/factor for men	Choosing active surveillance *I have no symptoms…it is a very small cancer. It’s not aggressive. So I decided that was the course for me. I would take active surveillance. I am not afraid of dying.* *At my age, there’s this notion that one could die before you die of prostate cancer, you die of other causes.* Staying on active surveillance *It’s been 7 years and I feel fine…as long as my numbers are good, my exams are good, I am going to stay on active surveillance*. *If I was ever going to have another biopsy, and there were shown major changes in the amount of cancer in the biopsy bits, and if the doctor suggested that well maybe we should consider an operation, I mean that is the kind of information you want to hear to help you make a decision on it.*
	Quality of life is an important consideration/factor for men	Choosing active surveillance • *I told him I am not interested in anything of [treatment]. It’s about quality of life…I don’t have symptoms now, so I am going to wait.* • *Treatment is scary, in terms of side effects on things…the side effects of the hormone drug I might have to take are huge. I think with radiation as well.* Choosing treatment • *If I live for another 35 years, I would like it to be a good quality I don’t want to deal with cancer…me? I would just do it [treatment]. So I said, ‘Don’t wait, let’s just do it.*
	There is a need to balance what is important to you	*Some of these treatments have pretty drastic after effects, ah, you would have to live with them. So you have to weigh it all out.* *You sort of balance the things about invasive surgery, you know, and what else is happening to your body from other causes. If you are pretty healthy then you want to stay that way. If it gets serious, then I will think of the alternatives.* *You kind of have to figure out what you need for surviving and what you need for quality of life. It just seemed to me that active surveillance gave me options. I did not have a lot of disease and, who knows, if I wait for treatment until I really need it, there could be other things available.*
Conversations with doctor(s) have significant influences on men in their decision-making about active surveillance	Conversations with doctors can be helpful or add to a patient’s distress	*I talked with the surgeon and really just had one option offered to me. I was not satisfied with that, I wanted to have all of my options explained. So I went to another doctor.* *To be frank with you, I had my GP tell me, ‘You know, it’s a long term thing. Don’t worry about it’…he told me not to worry about it. So I am taking him at face value and I am not overly concerned.* *So at this point, in talking with two urologists and a radiologist, I’ve decided, well, I think they helped me decide, that the thing to do was, ah, not to do anything too drastic but to maintain surveillance of the growth.* *I have to say I was a little apprehensive before I talked with Dr. X and the radiologist…I was quite impressed because they were quite conservative in their approach. That kind of reinforced my thinking because I was a little bit apprehensive about going through any kind of radiation treatment or something like that.* *There is no doubt about it, you turn to the physician and depend on what he is telling you what is right for your situation. He can certainly sway you one way of the other.*
	Confidence and trust are of importance	*What persuaded me most was the reaction of the medical staff. They didn’t seem to be overly excited about the whole thing.* *I talked with the first urologist and then with two other doctors, and I was sort of reassured. I mean I may need treatment sometime, that may happen, but for the moment I’ve decided to do surveillance* *He has high credentials. So you know he knows what he is doing…keeping up to date…pleasant....his reputation. Trusting the doctor is the answer.* *This guy has a way of talking with you. He explains. He shows you…but he leaves you, it’s your decision.* *I listen to him. I have been with him for more than 11 years. I do what he says, he knows better than me*. *I listened to what the doctors had to say. There were slight variations, but not much. There’s some consensus there.*
	The ideal process from a patient’s perspective allows for tailored discussion and reflection	*You have to help people prepare for what they are heading into.* *You need to give them an honest appraisal of their status. They can govern themselves…it is important that you let them know it is mild and slow progressing...it is a different type of cancer.* *Dissemination of information is very important on a direct basis…you give as much information as you can.* *The person [who goes on active surveillance] wants to know, if I go on this regime of just testing every so often, am I at risk? Will it get past the threshold of danger, you now, before we find out?...having time to act if something begins to change, is there time to act? That’s critical. That’s what makes action surveillance safe for us.*

#### Theme: an important decision is needed at a time of emotional upset and uncertainty

Consent for AS was recognized as an important decision that required careful consideration within a context of emotional upset and uncertainty. The emotional upheaval arose during the assessment of a diagnosis, in hearing the diagnosis, and upon request for a decision without sufficient information and understanding.

##### Obtaining a definite diagnosis can take time

Few participants had symptoms before their diagnosis. Most had been followed by their family doctors with regular PSA testing prior to observing a rise in levels. Subsequent referral to a surgeon or urologist usually resulted in a biopsy. The diagnosis was based on, at minimum, one biopsy result. However, it was not unusual for the men to undergo more than one test and to wait for various lengths of time for a definite diagnosis.

##### Hearing the definite diagnosis is a shock

Participants acknowledged that they were shocked and upset at hearing the PCa diagnosis, even though they understood that tests were performed to detect the disease. They indicated feeling numb, overwhelmed, frightened, and uncertain about the future, questioning whether the results were correct and why this had happened to them.

##### Having little information about PCa causes uncertainty

Many participants stated that they knew very little about PCa and its treatment at diagnosis. They did not understand what caused the disease, their risk of death, how the cancer was treated, what treatment options were available, or the impact of various treatment approaches. Those who knew something about the disease were drawing on experiences of family members or friends. Essentially, they perceived cancer as a fatal illness and experienced uncertainty about their situation and fear for their mortality.

##### Being told it is your decision to make was surprising

Many participants were dismayed when they discovered that they had the final say on their treatment. They had anticipated that the physician or surgeon would provide clear direction on which treatment option to pursue. They were surprised in the variety of options, feeling startled and, to some extent, frustrated when they realized that they had a significant role in the decision-making instead of principally complying with the best course of action from an expert. Many wondered on how to make a good decision without adequate information.

#### Theme: information is necessary on a number of topics before a decision can be made about AS

The participants cited the need for information as a critical element in making their decision about AS. Important components included understanding the information on a number of topics, searching for the information by oneself, and applying and detecting the relevant information to one’s own situation. Participants’ experiences varied across these components in terms of fulfilling their needs.

##### Information is needed on PCa, treatment options, benefits, side effects and AS

Participants listed a range of topics to comprehend before making an informed decision about AS. Understanding the disease, the various options for treatment, and the anticipated outcomes and side effects of each option were commonly cited. The men sought for knowledge of topics in general and an understanding of facts related to their own situation. In particular, they needed to understand AS and its intended benefits. Most had not heard of AS and had initially interpreted it as doing nothing to combat a feared disease.

##### There is a need to actively seek information

Once they had learned their diagnosis, participants felt that actively seeking information was critical for making an informed decision. For some, this step came naturally whereas others searched for information because they wanted to confirm the information from their physicians or felt that it was incomplete. Others wanted a second or third opinion, input from other cancer patients, or assistance in understanding what they were reading. A commonly held perspective was that one had to be self-reliant in educating themselves and could not solely rely upon the information received from health care professionals.

##### There is a need to check with different sources of information

Participants utilized different sources for their information search and were generally encouraged to do so by their physicians. The internet was the most cited resource, followed closely by the advice from health care professionals, family members and friends. Men found it helpful when cancer centres provided information packages or referrals to local PCa peer groups. Different sources offered input on different topics; while some provided factual information, others offered the opportunity for discussion, particularly among peers who had been through a similar situation.

##### It is important to sort out what information is relevant to the individual’s situation

Men expressed concern about the reliability of information from various sources, the amount of searching they had to do without guidance from health care professionals, the large amount of information available, and the wide variation in suggested treatment approaches for PCa. Participants expressed surprise and frustration on the lack of agreement between treatment guidelines and indicated the benefits of talking with a knowledgeable individual who could apply information that was specific to their own situation. Men found it helpful in knowing that they could take time to reflect on what they were learning and how it applied to them, and not feel pressured to make an immediate decision.

#### Theme: disease status and quality of life are important factors for men in deciding about AS

Disease status and quality of life emerged as the most important factors to consider in the decision for AS. Participants sought a wide range of information, but acknowledged that primary influences included the nature of their disease and the quality of life they wanted. These same factors applied for men who selected AS or those who elected to pursue interventional treatment, but the interpretation and application to a man’s situation varied. For example, age could be used to argue for pursuing immediate surgery (e.g., recover more quickly) or electing to wait (e.g., too young to live with potential impotence).

##### Various factors are taken into consideration for a final decision about AS

Participants described a range of factors they considered when deciding their course of action. These included understanding their disease and its treatment, their risk of death, AS and its intention, treatment side effects, age and health status (both now and later), the desire to be cancer-free, medical opinion, past experiences with cancer, family perspectives, and the emotional toll of living with cancer. Factors were prioritized differently based on the individual’s life experience and situation.

##### Disease status is an important consideration/factor for men

Men considered their disease status as a key factor when selecting either a course of AS or interventional treatment. The type and size of tumor and risk for death from the cancer were significant pieces of information. Prior to their diagnosis, the participants had not understood that low grade or slow growing PCa did not require interventional treatment. Most had not heard of AS and were introduced to the idea as an option for low grade disease by their physicians. Although some confused interchangeably the terms ‘watchful waiting’, ‘routine follow-up care’ post-surgery and AS, participants described AS as appropriate for low grade disease; they understood the delay in treatment and its side effects while being closely monitored, and that interventional treatment was applied when it became necessary. Treatment was described as necessary if the disease progressed and the physician indicated that action was required. However, waiting offered the possibility of new treatment approaches being available at a future date as well as avoidance of an operation and disturbing side effects. Comfort with an AS approach was based on the idea of being closely monitored with reliable tests, and acting in a timely fashion when required.

##### Quality of life is an important consideration/factor for men

The issue of side effects emerged prominently in discussions about quality of life. Both radiation and surgery for PCa were seen as having significant side effects with potential to impact a person’s relationships, sexuality, functioning, and emotional well-being. Avoiding side effects was seen as desirable by participants and figured highly in their decision-making about AS. Once they were reassured that their health was not at greater risk on AS with access to treatment in a timely fashion if required, men viewed AS as a preferred option. The subsequent challenge was living with the idea of cancer in their bodies and not dwelling on that fact on a daily basis. Given that most of the participants were not experiencing symptoms, avoiding side effects became a clear choice for quality of life.

##### There is a need to balance what is important to you

Participants described how they came to realize that the decision about treatment was largely their responsibility. However, this realization emerged only after seeking information and discussion with health care professionals, family members and others. In the final analysis, they needed to identify and give due consideration to what was important to them, and balance various considerations for their life situation. In particular, the men reflected that the idea of AS was different from the public cancer messages about early diagnosis and prompt treatment. They, alongside family and friends, had to come to terms with understanding this difference. More often than not, family and friends were encouraging the men to the treat the cancer.

#### Theme: conversations with doctors have significant influences on men in their decision–making about AS

Almost without exception, participants spoke of conversations with doctors as significant for them and in their decision-making. Many men relied on the physician/surgeon for clear direction on which treatment option to pursue. This included helping them understand the benefits and consequences of various treatments, and what the best approach would be for them, given their situation.

##### Conversations with doctors can be helpful or add to a patient’s distress

Participants described wide variation in the nature of conversations with physicians. Variation existed in the duration of the conversations, the clarity of information provided, the personal care and concern that was expressed, consistency of information from different doctors, who was involved in the conversational exchange, the number of treatment options and specific effects discussed, and the active encouragement to seek other opinions. While some participants were entirely satisfied with the exchange and felt well informed and supported afterwards, others found these conversations were less than helpful and experienced heightened frustration and uncertainty.

##### Confidence and trust are of importance

Participants expressed a high sense of confidence and trust in the physicians and their viewpoints. For many, this confidence emerged from conversations held, the relevance and clarity of the information provided by the physician, opportunities to openly question and discuss test results and options, and the attention paid to the men’s individual preferences. For others, the confidence emerged from having known the doctor over many years or knowing the physician’s reputation.

##### The ideal process from a patient’s perspective allows for tailored discussion and reflection

Participants readily identified important characteristics of an ideal process for holding conversations about AS. These included easy access to information, having time for conversational exchange, providing clear and honest information, providing information relevant to the individual’s situation, delivering the information with sensitivity and compassion, including family members, supporting the conversation with written information, and providing reliable web-site addresses for personal follow-up. Clearly, participants viewed broad access to information as an important feature, but emphasized that information should be tailored or applied to the individual’s particular situation, preferences, and values. Access to relevant understandable information was of paramount importance in assisting men with their decisions and in preparing for what was ahead.

## Discussion

In this qualitative study, a wide-ranging discussion about patients’ decision-making process regarding AS showed that selecting AS was not an isolated decision but involved the entire experience of confronting the reality of having cancer.

A number of participants’ perspectives have been reported previously: emotional upheaval at diagnosis and surprise at the PSA elevation with no symptoms (reviewed in [10, 11]); lack of knowledge about PCa and its treatment, and lessening of anxiety once men understood more about their situation and available choices [12] (reviewed in [11, 13, 14]); influence of emotional anxiety on treatment decision-making [15, 16]; variation in preferences for involvement in decision-making, including the desire of some men for a passive role [17–19]; the need to search for information and the frustration of searching on their own [19]; the need for support from others (reviewed in [20]); the participants’ view about the significance of the physician’s role in the decision-making process [12, 18, 19, 21, 22] (reviewed in [11, 13, 20]); and the differences in the provision of information from different cancer programs or providers [23].

Unique to this study was the insight into the ways men approached decision-making regarding AS. At the onset of their experience, they were unfamiliar with AS and following a course of AS ran counter to the men’s original preconceptions about cancer and treatment. They identified a need to shift their thinking about what actions to take. Once men had relevant information, understood the actual degree of risk for themselves, and perceived the opportunity to avoid treatment side effects, some became more open to the idea of AS.

Understanding that their disease was low risk, combined with the notion of avoiding treatment side effects, were strong incentives for those who opted for AS. Enduring treatment side effects was perceived as reducing their quality of life. This aligns with a questionnaire analysis [15] and a study reporting analysis of internet conversations showing an increased awareness of quality of life and associated comfort with AS [24]. In contrast to participants in a reported phone survey analysis, patients from our focus groups felt well-informed on the possible side effects of treatments [25]. They also emphasized the comfort in being closely monitored, as reported in other studies [12, 21]. Close monitoring would allow for future treatment intervention in a timely fashion, if necessary. This idea was important for the men and contributed to their ease in handling the sense of risk.

For those who opted not to follow a course of AS, many talked about their capacity to undergo immediate treatment, based on their age or physical status, or feeling a pressure to deal with the cancer, which emerged from their internal sense of risk from the disease or from commentary by family members. The desire to be cancer-free or to reduce their risk or on-going worry, influenced their final decision to pursue surgery or radiation treatment. These findings also aligned with other recent reports [12, 15, 21, 26, 27]. Anxiety was a reason not to adhere initially to AS but was also a factor that influenced the decision for a radical treatment after a period of time on AS (reviewed in [20, 28]).

Most participants consulted a number of individuals, either for information or for support, while deciding about AS. Variation existed in whom they consulted, what input they sought, and the helpfulness of responses they received. In particular, family members and friends were consulted frequently. However, they were often unaware of PCa treatment options and had to adjust their ideas about the low risk nature of the disease, as did the men. Other health care professionals, including family physicians, were also consulted regarding AS. Participants found that these practitioners did not always have the necessary information on AS. Finally, a few men had the opportunity to meet with a support group for men diagnosed with PCa; those that attended meetings found the conversations with peers informative.

Ultimately, the role of the physician in the decision-making process was significant to the participants; they referred to this person for advice and direction. Confidence and trust was engendered primarily through the manner in which conversations unfolded and the way questions were answered. Fundamentally, men sought for clarity in information and forthright conversations about their risks from PCa (now and in the future), and time for discussion about their unique situation. Inconsistency in information from one practitioner to another, or one treatment guideline to another, was distressing for these men, as were hurried conversations with medical technical language. These findings are in line with those reported previously where the increased number of visits to the specialist rendered the treatment decision more difficult for men [25] and raised more concerns by patients in regards to the unbiased recommendation of treatment by their specialists [24].

### Implications for practice and future research

With one exception (the Thunder Bay group), all focus groups were held in metropolitan academic centres where PCa care was delivered within specialized clinical programs. Nonetheless, men identified variation in their access to relevant, meaningful information about PCa, treatment options, and AS. This raises questions about the process for providing information to men and their family members and emphasizes the need for standardization across the country. Ultimately, the content should be clear and comprehensive, and the delivery should include multiple types of formats and easy access. Where possible, the opportunity to discuss the information in relevance to an individual’s situation could be deemed valuable to those interested.

We isolated several factors that men took into consideration when deciding their PCa treatment. Conversations with clinicians need to include dialogue about these factors with as much clarity as possible. In particular, tools for decision-making (i.e., decision boards) around AS could be designed and tested. This concept was assessed and deemed helpful by low risk PCa patients [29]. However, based on a systemic review, the majority of decision-aid tools are lacking important information for PCa treatment decisions or do not provide sufficient elements to favor shared decision-making by both the patient and the health care provider (reviewed in [30–32]). Organizing the opportunity for more than one conversation with time to make decisions would likely be of benefit to men, and allow clarification of facts and anticipated implications of one course of action over another. Follow-up studies could include interrogating the universality of specific findings of our qualitative study, using a more quantitative questionnaire-based approach, to more fully understand the patient-centered decision making process around active surveillance.
